# Effects of Methamphetamine Withdrawal on the Volume and pH of Stimulated Saliva

**DOI:** 10.30476/DENTJODS.2021.87248.1244

**Published:** 2022-06

**Authors:** Haleh Zokaee, Shima Fathi, Hossein Golalipour, Fatemeh Mirzaei

**Affiliations:** 1 Dept. of Oral and Maxillofacial Medicine, Dental Research Center, Golestan University of Medical Sciences, Gorgan, Iran; 2 Oral and Maxillofacial Medicine Specialist, Hamedan, Iran; 3 Dentistry Student, Student Research Committee, Golestan University of Medical Sciences, Gorgan, Iran

**Keywords:** Methamphetamine, Withdrawal, Saliva

## Abstract

**Statement of the Problem:**

As a stimulant drug of the central nervous system, methamphetamine reduces salivary secretion by stimulating inhibitory α2-receptors in the sympathetic system. The acidity of this substance reduces the salivary pH and causes severe dental caries and erosion in the cervical region of teeth, appearing as "meth mouth".

**Purpose:**

This study aimed to determine the effects of methamphetamine withdrawal on the volume and pH of stimulated saliva in patients under treatment at rehabilitation centers.

**Materials and Method:**

This descriptive cross-sectional study was conducted on a total of 20 individuals at the rehabilitation centers of Gorgan, Iran. The volume and pH of stimulated saliva were measured at three intervals: before withdrawal, four days after withdrawal, and 30 days after withdrawal. Data analysis was performed using ANOVA test in SPSS.

**Results:**

The results showed a significant difference in the mean volume of saliva before and after withdrawal (*p*< 0.05). Thirty days after withdrawal, the mean volume of saliva was significantly different from the mean volume after four days (*p*< 0.05). Moreover, the mean pH of saliva after withdrawal was significantly different from the mean pH before withdrawal (*p*< 0.001). On the other hand, the mean salivary pH at 30 days after withdrawal was not significantly different from the mean pH on the fourth day after withdrawal (*p*> 0.05).

**Conclusion:**

It seems that methamphetamine withdrawal influences the volume and pH of stimulated saliva in patients under treatment at rehabilitation centers.

## Introduction

Saliva is essential for gastrointestinal function and protection of the oral cavity. Xerostomia, defined as a reduction in the stimulated saliva to less than 0.7 ml/min [ 1 ], has always been a major concern for dentists as it significantly increases the risk of tooth decay, erosion, and periodontal disease [ 2 ]. Many etiological factors have been described for xerostomia, the most common of which is drug use. Overall, stimulant drugs can be introduced as the cause of xerostomia for several pathophysiologic reasons [ 1 , 3 ]. In general, addictive substances are divided into two groups: opiates and central nervous system (CNS) psychostimulants. Opiates reduce pain and cause slight sedation and euphoria. These substances directly affect the CNS, resulting in nausea, vomiting, pain relief, euphoria and sedation. In the event of substance abuse, respiratory depression occurs due to decreased brainstem sensitivity to carbon dioxide (CO_2_). Toxic reactions in case of drug abuse may appear as bradycardia, temperature drop, coma and even death [ 1 - 2 , 4 ].

An addict is defined as a person, who has experienced the effects of substance abuse (narcotics or CNS stimulants) and withdrawal syndromes with positive results on multi-drug laboratory tests [ 5 ]. Heroin, morphine, and codeine are natural drugs, Dilaudid (hydromorphone) and Oxycodone are semisynthetic drugs, and meperidine, propoxyphene, methadone and pethidine are synthetic drugs. Amphetamines, including Desoxyn, Dexedrine, and Pemoline, are in the class of CNS stimulants. They are prescribed for the treatment of hyperactivity, Parkinson's disease, obesity, and headaches, which do not respond to common medications. 

Methamphetamine is a form of psychoactive amphetamine, which is very strong and addictive and is referred to as "Zip", "Go", "Crank", and "Speed" in the Western market [ 1 ]. This substance was synthesized for the first time in 1919. Considering its stimulant properties, it became increasingly popular among soldiers in World War II. Abuse of methamphetamine was common among athletes and students until 1595 when it was banned by the Food and Drug Administration (FDA) [ 6 ]. Methamphetamine can be smoked, inhaled (snorted), injected, or orally ingested. In Iran, it is known under trade names, such as "Glass", "Shabu", "Chalk" and "Crystal" [ 7 ]. The acute effects of methamphetamine on the sympathetic nervous system increase blood pressure, body temperature, and respiratory rate and increase the risk of stenosis, tachycardia, and pupil dilation. Besides its effects on the peripheral nervous system, it is associated with serious damage to the mouth, jaws, and face, including traumas following overdose and pathological changes [ 2 , 8 ].

According to current statistics from Iran, methamphetamine abuse has significantly increased from 6% in 2009 to 20% in 2011, and this figure is expected to increase in the coming years [ 7 ]. Long-term abuse of methamphetamine causes bruxism, xerostomia, severe caries, and dental erosion, known as "meth mouth" in addicts [ 2 , 9 ]. Xerostomia is developed through two mechanisms. In the first mechanism, α2-receptors are stimulated in the CNS, which in turn triggers the sympathetic system, with inhibitory effects on saliva secretion from the salivary glands (resulting in xerostomia as a clinical outcome) [ 2 , 10 ]. In the second mechanism, after methamphetamine abuse, the patient experiences excitement, hyperactivity, and loss of appetite followed by reduced absorption of water and food, which causes dehydration and reduced saliva secretion [ 2 , 6 , 11 ].

Under normal conditions, the pH of the mouth is six to seven on average [ 1 ]. In methamphetamine addicts, reduced salivary secretion, as well as methamphetamine acidity, decreases mouth pH and causes severe caries and dental erosion in the cervical area [ 2 ]. A study by Okubo *et al*. [ 12 ] showed that methamphetamine withdrawal activates the pituitary adenylate cyclase-activating polypeptide-diazepam binding inhibitor (PACAP-DBI) pathway in mice, thereby reducing salivary secretion. A study by Haile *et al*. [ 10 ] suggested that the activity of the noradrenergic system is significantly related to the activity of salivary alpha-amylase and methamphetamine abuse. 

In South Africa, Grobler *et al*. [ 13 ] conducted a study on the pH of various types of methamphetamine. In their study, 29 different types of methamphetamine were collected. The pH range in the samples was 3.02 to 7.03 (mean= 5). Moreover, 72% of samples had a lower pH than the salivary pH. This study concluded that most available methamphetamines in the market had a low pH; however, this pH level could not directly cause damage to teeth in the event of reduced saliva production.

Despite several studies on oral manifestations, xerostomia, pH reduction, and direct effects of methamphetamine abuse, there are limited studies on the quantity of saliva and the side effects of methamphetamine withdrawal and reversible xerostomia [ 14 - 16 ]. So far, only one study [ 12 ] has examined the effects of addiction treatment on saliva secretion changes in an animal model; therefore, further research is needed to generalize the results to humans. Moreover, no study has determined the amount of time required for reversing salivary function to normal. Therefore, the present study aimed to determine the effects of methamphetamine withdrawal on the volume and pH of stimulated saliva in patients under treatment at rehabilitation centers.

## Materials and Method

In this analytical cross-sectional study, patients admitted for methamphetamine withdrawal to the rehabilitation centers of Gorgan, Iran, were recruited. Local Ethical Committee clearance was obtained (IR.GOUMS.REC. 1394.203). The consent form was completed and signed by each patient. All patients with confirmed methamphetamine addiction (based on multi-drug tests), receiving treatment at the rehabilitation centers, were included in the study. The exclusion criteria were as defined as (1) addiction to other substances along with methamphetamine, (2) use of drugs, which decrease saliva production such as tricyclic antidepressants (TCAs), anticholinergicdrugs, and selective serotonin reuptake inhibitor (SSRI), (3) pathologies of the salivary glands, such as stones and obstruction, fibrosis and sclerosis,(4) connective tissue diseases such as Sjögren syndrome and Mikulicz's disease and (5) reuse of amphetamine stimulants during withdrawal.

The mean amount of saliva in the parotid, submandibular, and sublingual salivary glands was estimated at 115.4±6.7, 106±8.6 and 47.6±6.3μl/h, respectively in the pre-withdrawal stage. On the other hand, in the post-withdrawal stage, the level of saliva was 63.6±12.1, 56.5±10.7, and 25.8±3.2 μl/h, respectively. By assuming a minimum of 20 units of change and standard deviations of 8.6 and 10.7, at least 6 samples were required, based on the following formula (95% confidence level; power, 80%): 

$N=\frac{{\left(z_{1-\frac{\alpha }{2}}+z_{\mathrm{1-\beta}}\right)}^{2}(S_{1}^{2}+S_{2}^{2})}{{\left({µ}_{1}-{µ}_{2}\right)}^{2}}$

In the present study, 20 patients were monitored for more precision, considering the differences between human and animal models. As the subjects were not allowed to leave the centers, urine sampling was performed at the center in glass containers. The samples were then transferred to the laboratory of 5 Azar Hospital of Gorgan and tested by a laboratory expert using a multi-drug test. Individuals, who had non-methamphetamine contents in their urine, were excluded, while those with only methamphetamine in their urine samples were included in the study. Each individual was studied at three intervals (before withdrawal, 4 days after withdrawal, and 30 days after withdrawal) in terms of the volume and pH of stimulated saliva. In order to collect the salivary samples, the participants first were asked to chew 1 g of paraffin wax, and after 1 minute, the saliva was drained off in a 25mL container. After measuring the saliva volume, the saliva pH of each individual was measured by a pen type pH meter (AZ8689, AZ Instrument Corporation, Taiwan) with a precision of 0.01. Finally, the pH meter was rinsed with distilled water and disinfected by a Deconex spray. 

Data analysis was performed using SPSS software version 16. The normality of variables was assessed by Shapiro-Wilk test. Since variables were distributed normally, they were assessed by ANOVA and Bonferroni test. Pearson correlation rank was used for linear relation assessment of volume and pH of saliva. Descriptive statistics (mean, standard deviation, frequency, and percentage) were done. The level of significance was set at 0.05.

## Results

In this study, 20 male patients were included and age was not considered as a determinant variable. According to Table 1, the mean salivary pH was 5.48±0.54 in the pre-withdrawal stage, 7.03±0.54 at 4 days after withdrawal, and 6.97±0.52 at 1 month after withdrawal. According to the repeated measures ANOVA test, the mean values were significantly different (*p*< 0.001).

**Table 1:** Comparison of the mean and standard deviation of saliva pH at three intervals

Stage	Mean	Standard deviation	pH range
Pre-withdrawal	5.48	0.54	4.23-6.78
4 days after withdrawal	7.03	0.54	5.94-7.82
30 days after withdrawal	6.97	0.52	5.82-7.63

According to Table 2, two-by-two comparison of pH at different intervals showed a significant difference in the mean pH before withdrawal and 4 days after withdrawal (*p*< 0.05). In addition, there was a significant difference in the mean pH at 30 days after withdrawal and before withdrawal (*p*< 0.05). However, there was no significant difference in the mean pH at 4 days after withdrawal and 30 days after withdrawal (*p*> 0.05).

**Table 2:** Two-by-two comparison of salivary pH at 3 intervals

Stage	Mean	Standard deviation	*p*Value
Before withdrawal and 4 days after withdrawal	1.55	0.15	<0.001
Before withdrawal and 30 days after withdrawal	1.49	0.15	<0.001
4 days after withdrawal and 30 days after withdrawal	0.05	0.05	1

**Figure 1:**
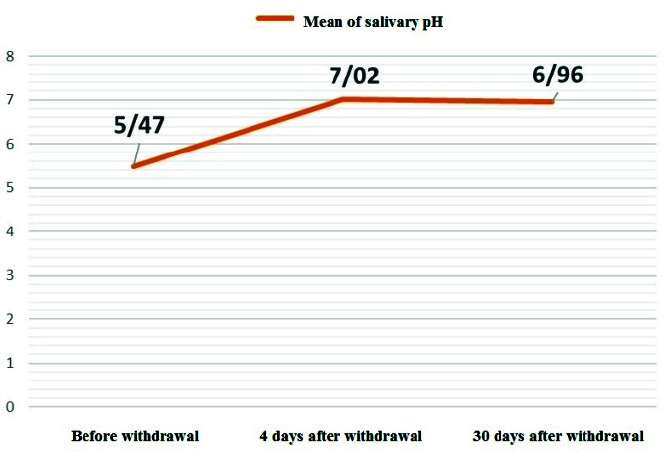
Comparison of salivary pH at three intervals

Figure 1 shows the results of mean comparison of salivary pH before withdrawal, 4 days after withdrawal and 30 days after withdrawal in the patients. 

According to Table 3, the mean saliva volume was 1.45±0.44 in the pre-withdrawal stage, 90.2±0.72 on the 4th day after withdrawal, and 79±3.47on the 30th day after withdrawal; according to the ANOVA results, the mean values were significantly different (*p*< 0.05).

**Table 3:** The mean and standard deviation of stimulated saliva volume in different stages (values ​​ presented in mL)

Stage	Mean	Standard deviation	Range
Pre-withdrawal	1.45	0.64	0.4-2.9
4 days after withdrawal	2.90	0.76	1.7-4.2
30 days after withdrawal	3.47	0.79	2.1-5

According to Table 4, two-by-two comparison of saliva volume at different intervals showed a significant difference in the mean stimulated saliva before and after withdrawal (*p*< 0.05).

**Table 4:** Two-by-two comparison of saliva volume at three intervals

Stage	Mean	Standard deviation	*p*Value
Before withdrawal and 4 days after withdrawal	1.45	0.15	<0.001
Before withdrawal and 30 days after withdrawal	2.02	0.19	<0.001
4 days after withdrawal and 30 days after withdrawal	0.57	0.13	<0.001

**Figure 2:**
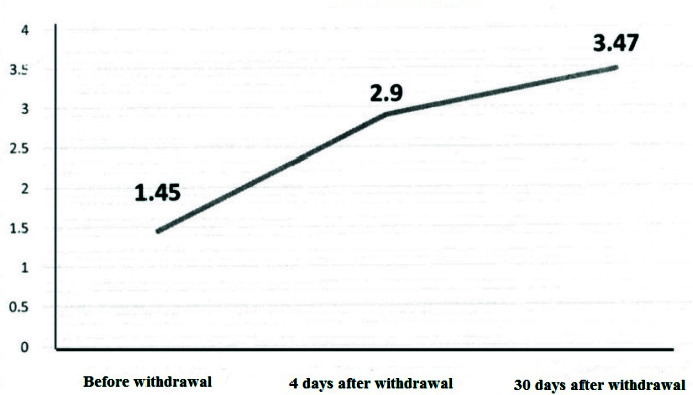
Comparison of the mean saliva volume at three intervals

The results also showed a significant difference in the mean saliva volume on the 4th day after withdrawal and 30th day after withdrawal (*p*< 0.05). Figure 2 presents the results of mean comparison of salivary volume before withdrawal, 4 days after withdrawal, and 30 days after withdrawal in the studied patients.

## Discussion

This is the only study conducted in this area, performed in an animal model, and there is no similar study on a human population. This study was conducted on a total of 20 patients under treatment at the rehabilitation centers of Gorgan, Iran.

The volume and pH of stimulated saliva were measured at 3 intervals including before withdrawal, 4 days after withdrawal and 30 days after withdrawal. The results showed a significant difference in the mean volume of stimulated saliva after and before the withdrawal. This finding is in agreement with the results of a study by Haile *et al*. [ 10 ], which showed that long-term methamphetamine abuse induced the sympathetic system activity and stimulated inhibitory α2-receptors, resulting in a reduction of saliva volume. In addition, some studies have shown that methamphetamine abuse reduces appetite and increases energy and physical activity, causing a decline in saliva volume [ 2 , 11 ].

The half-life of methamphetamine in the body is 12 hours, and its effect on the body significantly reduces after 24 to 48 hours [ 4 ]. Therefore, the effect of this substance on the body diminished in the second and third stages of sampling, and the CNS was no longer in the stimulated status, which in turn triggered salivary secretion and increased the amount of stimulated saliva. The results of the study also explained the increase in saliva volume at 4 days after withdrawal, compared to the pre-withdrawal stage.

In the post-withdrawal stages, there was a significant difference in the mean saliva volume at 4 and 30 days after withdrawal. In previous studies on the neurophysiologic effects of methamphetamine, the noradrenergic effects on the CNS were observed up to 1 month after withdrawal; accordingly, the third sampling stage was described as 30 days post-withdrawal [ 5 , 11 ]. Since regulation of major salivary glands and saliva volume is influenced by the function of the peripheral sympathetic nervous system, in the early days of withdrawal, stimulation of the sympathetic system is not completely eliminated and the increase in saliva volume was less significant on day 4, compared to day 30 after withdrawal [ 2 , 10 ]. According to the literature, methamphetamine withdrawal is basically divided into acute and subacute phases. In the acute phase, which is the first 7 to 10 days after withdrawal, the patient is agitated and suffers from nausea, diarrhea and vomiting [ 17 ]. Diarrhea and vomiting cause dehydration and subsequently reduce saliva production. In the subacute phase, symptoms of diarrhea and vomiting improve, resulting in an increase in saliva production, which can also explain the lower level of saliva on day 4, compared to day 30 after withdrawal [ 17 - 18 ].

Okubo *et al*. [ 12 ] showed that binding of diazepam binding inhibitor (DBI) to peripheral benzodiazepine receptor (PBR) produces a neurosteroid, called pregnenolone (PRG) in the mitochondria, which can activate gamma-aminobutyric acid (GABA) receptors. GABA and benzodiazepine receptors mediate the inhibitory mechanisms of the salivary glands.

Methamphetamine withdrawal is considered a type of stress, which can activate steroid biosynthesis through the PACAP-DBI pathway in the salivary glands and reduce saliva production. In other words, stress can produce steroid hormones as autocrine and decrease salivary secretion by activating GABA receptors in the salivary glands [ 12 ]. These observations are consistent with the results of the present study, which showed that the highest level of stress associated with methamphetamine withdrawal occurred within the first week. By generalizing the results of studies on mice to humans, the lower saliva production on the 4th day compared to day 30 can be explained. 

In the present study, the mean salivary pH was 48.5 before withdrawal, 7.03 at 4 days after withdrawal, and 6.97 at 30 days after withdrawal; an ascending trend was reported on the 4th day after withdrawal. Moreover, there was a significant difference in the mean salivary pH before and after withdrawal. 

Following methamphetamine use, appetite is reduced and consumption of sweet substances and drinks is increased as a result of more glucose requirements of the brain for more activity, as methamphetamine is a CNS stimulant. Therefore, the patient's desire for major meals is decreased, while appetite for carbonated beverages containing simple sugars is increased, which in turn decreases salivary pH due to a high-carbohydrate diet. After methamphetamine withdrawal, due to the reduced consumption of sweet substances and drinks, the oral pH is expected to reach the normal level [ 6 , 11 ].

According to a study by De-Curolis *et al*. [ 2 ], acidity of methamphetamine decreases salivary pH, which can explain the increase in salivary pH after withdrawal. However, in another study, acidity of methamphetamine is not considered an important factor in the reduction of salivary pH [ 6 ]. The mean salivary pH was not significantly different on days 4 and 30 after withdrawal. This finding cannot be explained, as data on the effects of methamphetamine acidity on salivary pH were inconsistent and the diets were not synchronized. Due to limitations of time and facilities, it was not possible to monitor patients under treatment for a longer period or to increase the sampling frequency. 

Since sampling was not performed in a single period, dietary control, and complete elimination of the effects of nutrition on saliva volume and pH were not possible. To the best of our knowledge, there was no related study on fast effect of withdrawal on saliva and its components; so further studies in this field should be undertaken.

## Conclusion

It seems that methamphetamine withdrawal affects the volume and pH of stimulated saliva in patients under treatment at rehabilitation centers. 

## Conflict of Interest

The authors declare no conflict of interest.
